# Supplementary figures and images for: Disulfide bridge-dependent dimerization triggers FGF2 membrane translocation into the extracellular space
Source: eLife. 2024 Jan 22;12:RP88579. doi: 10.7554/eLife.88579 (PMC10945597; doi:10.7554/eLife.88579)

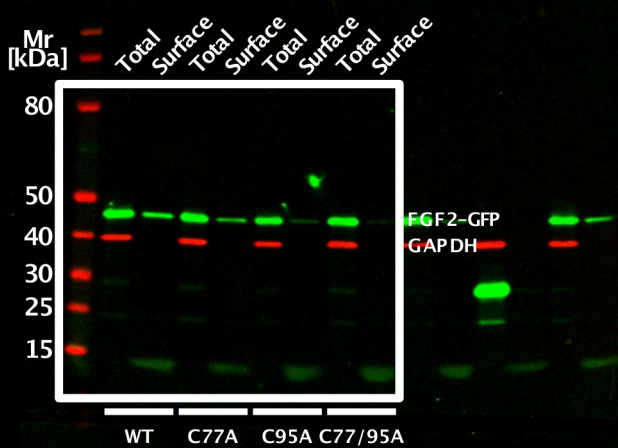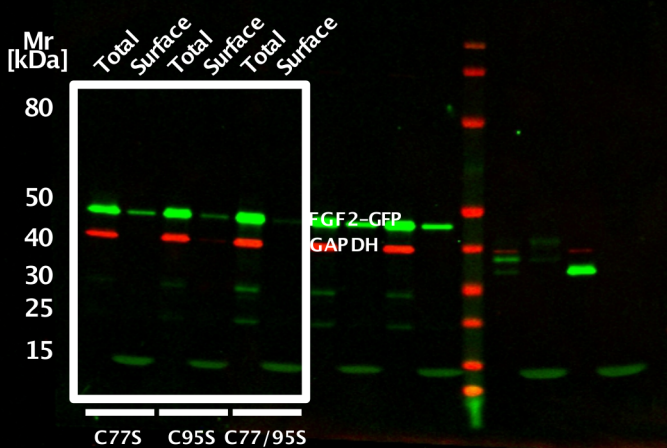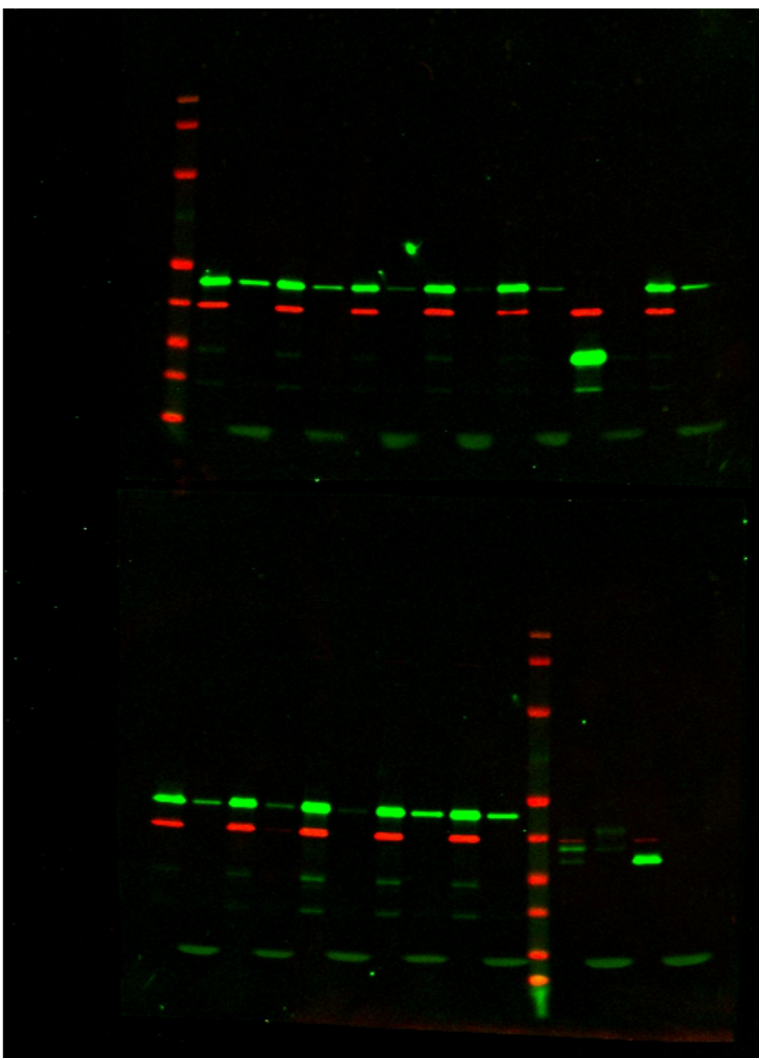

Supplement: Figure 1—source data 2. [file elife-88579-fig1-data2.zip › Figure 1ΓÇôSource Data 2.pdf]

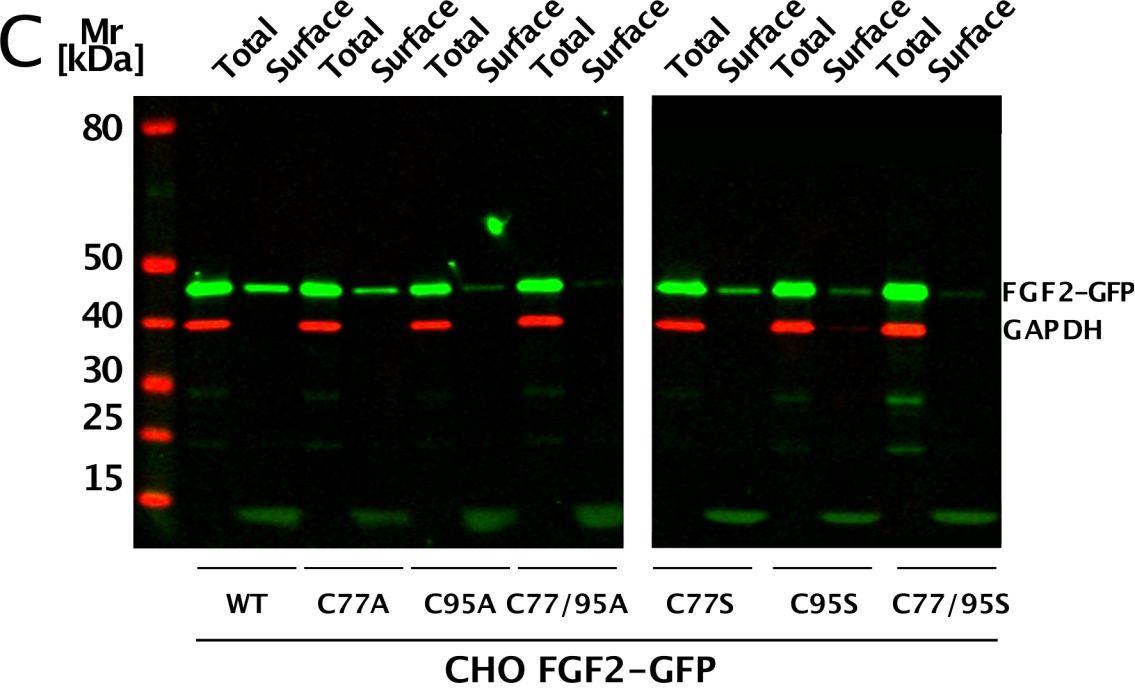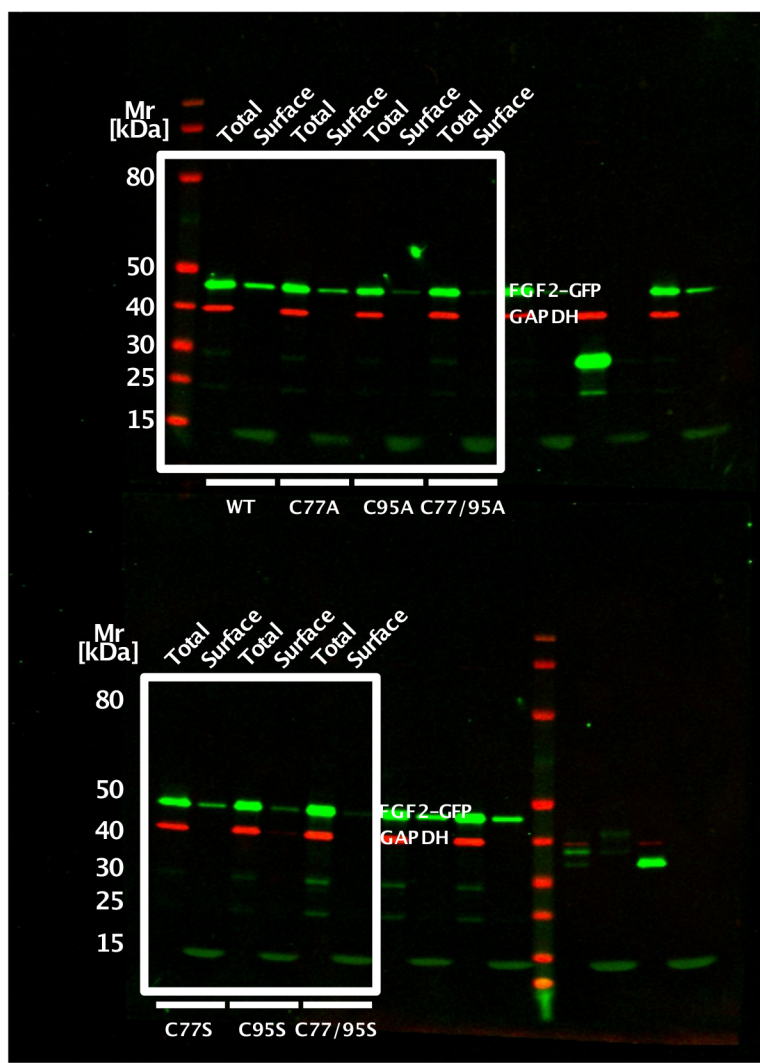

Supplement: Figure 1—source data 3. [file elife-88579-fig1-data3.zip › Figure 1ΓÇôSource Data 3.pdf]

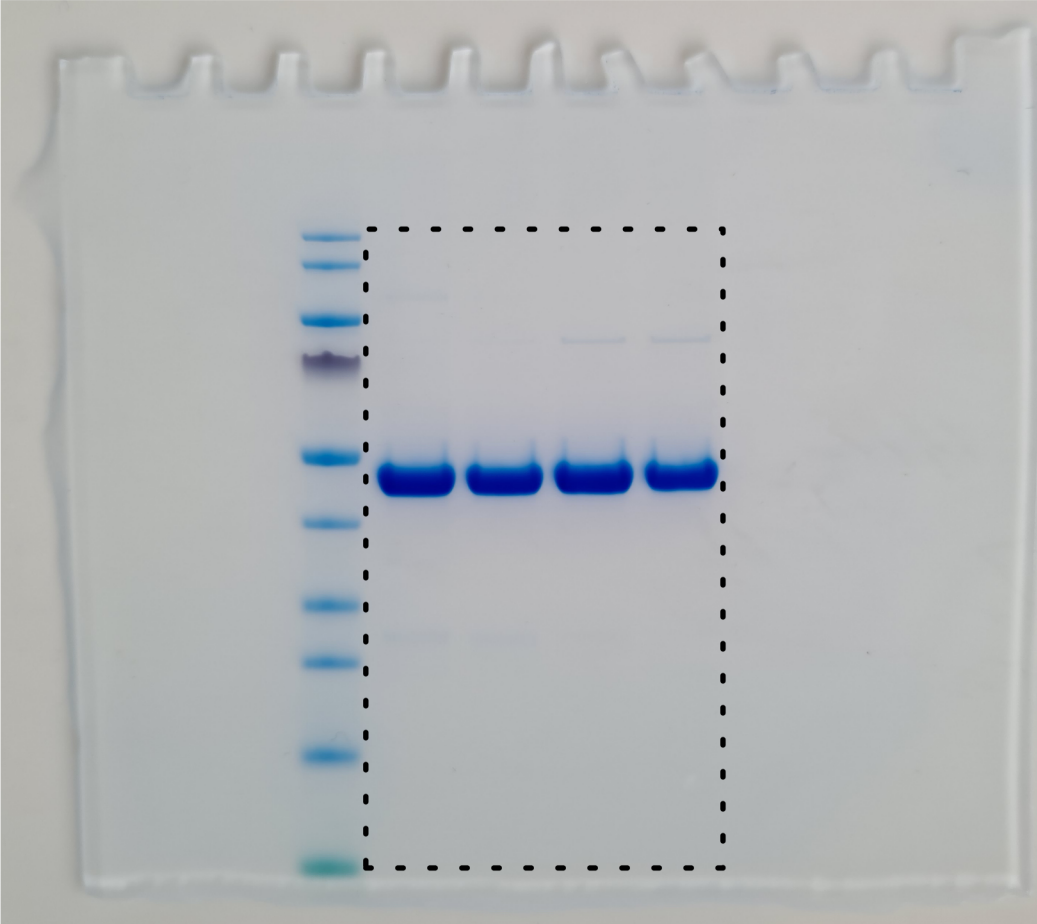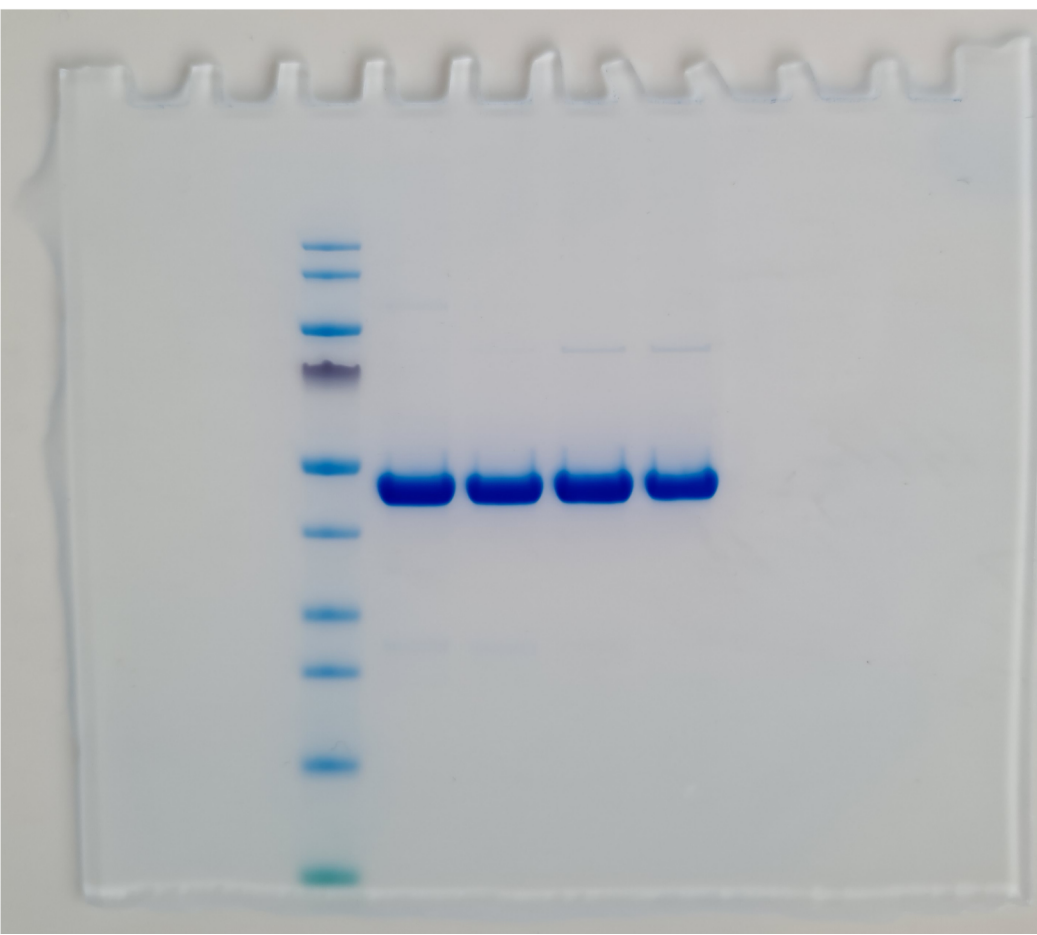

Supplement: Figure 2—source data 2. [file elife-88579-fig2-data2.zip › Figure 2ΓÇôSource Data 2.pdf]

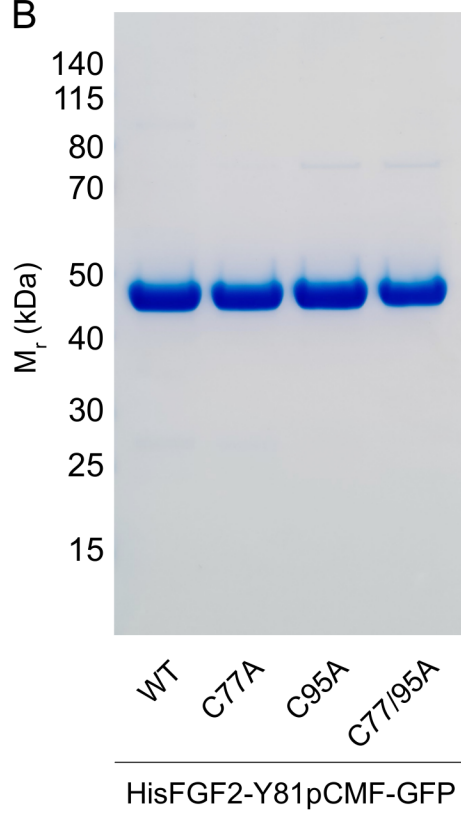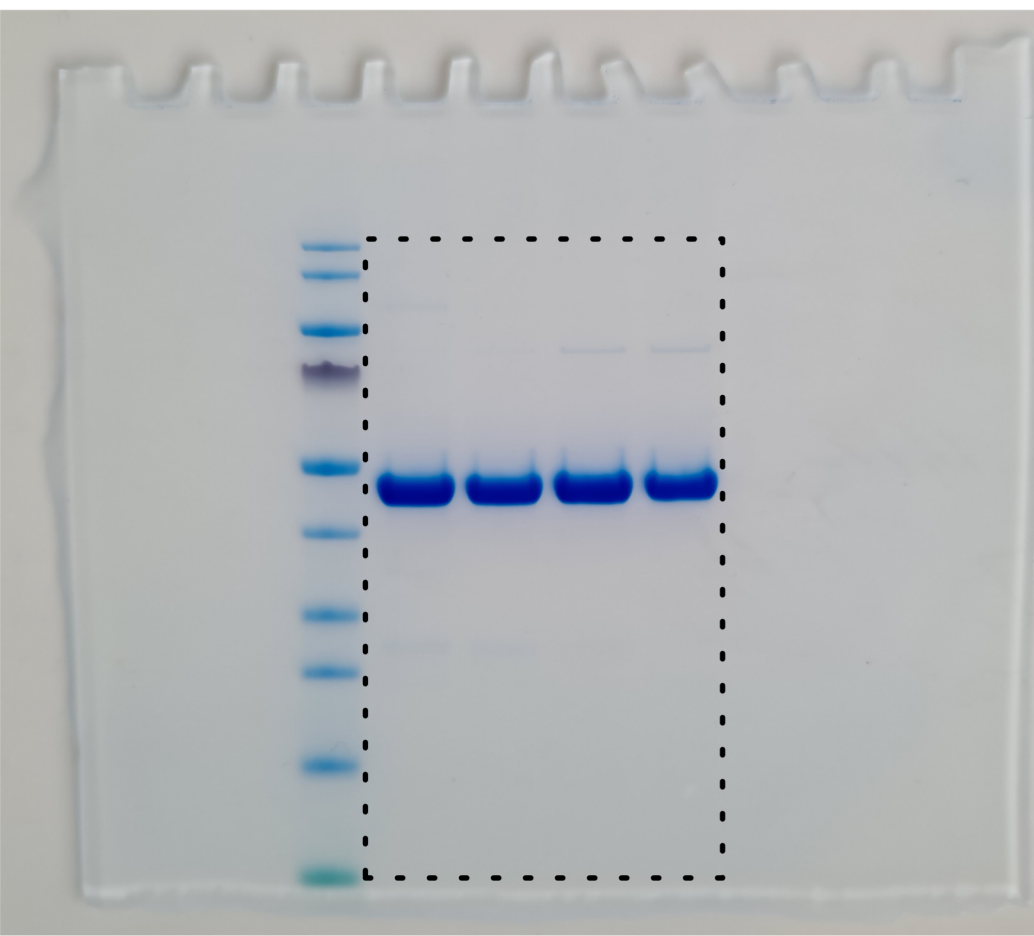

Supplement: Figure 2—source data 3. [file elife-88579-fig2-data3.zip › Figure 2ΓÇôSource Data 3.pdf]

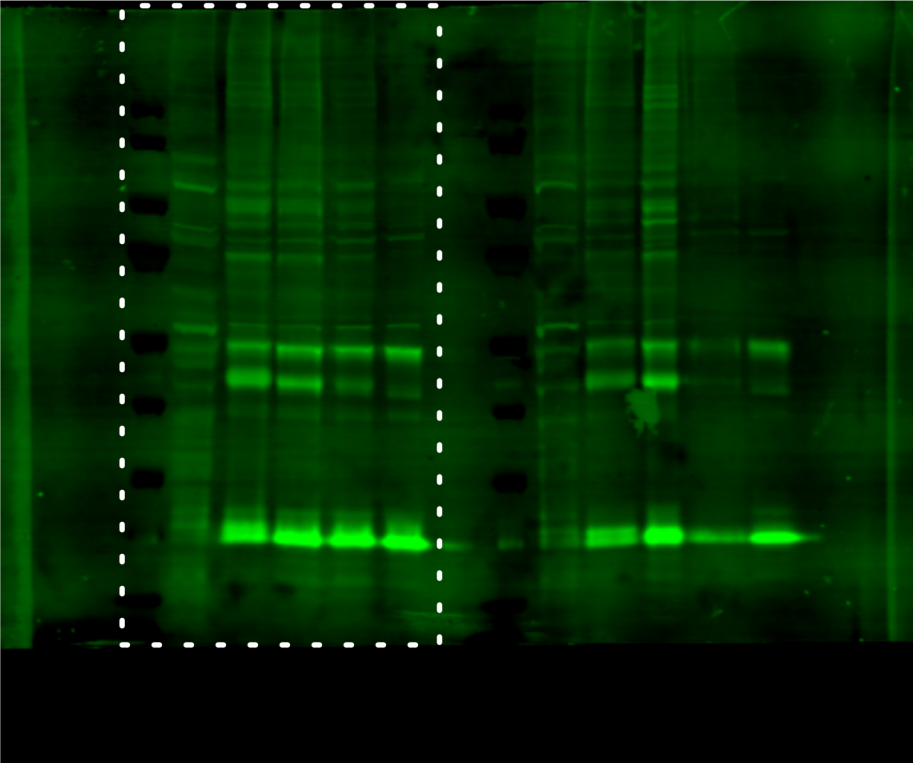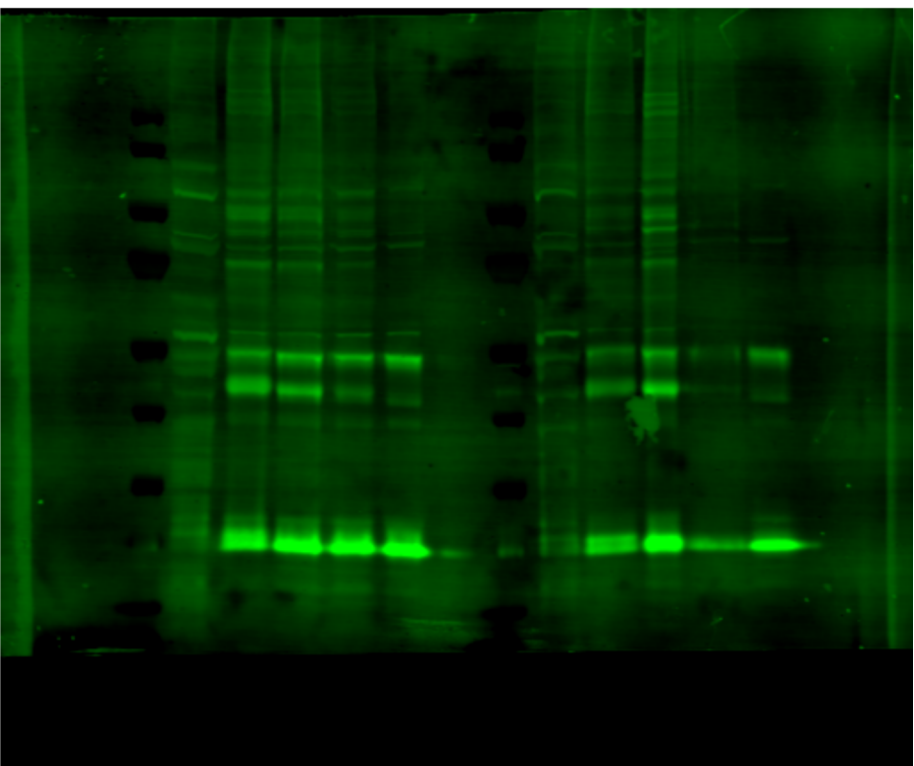

Supplement: Figure 3—source data 2. [file elife-88579-fig3-data2.zip › Figure 3ΓÇôSource Data 2.pdf]

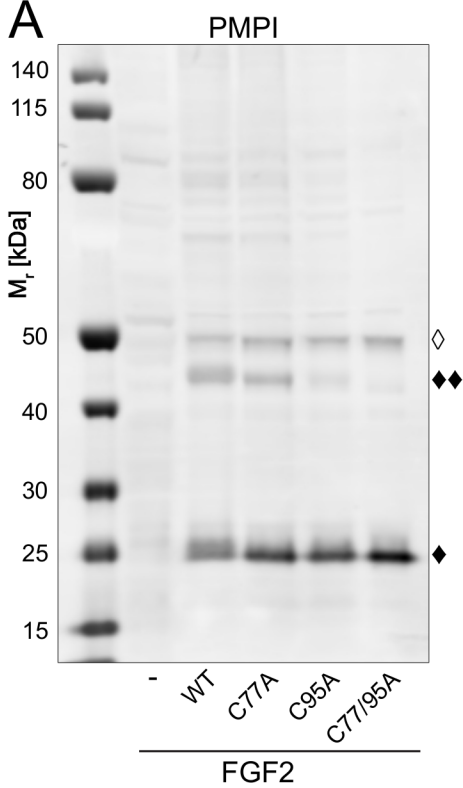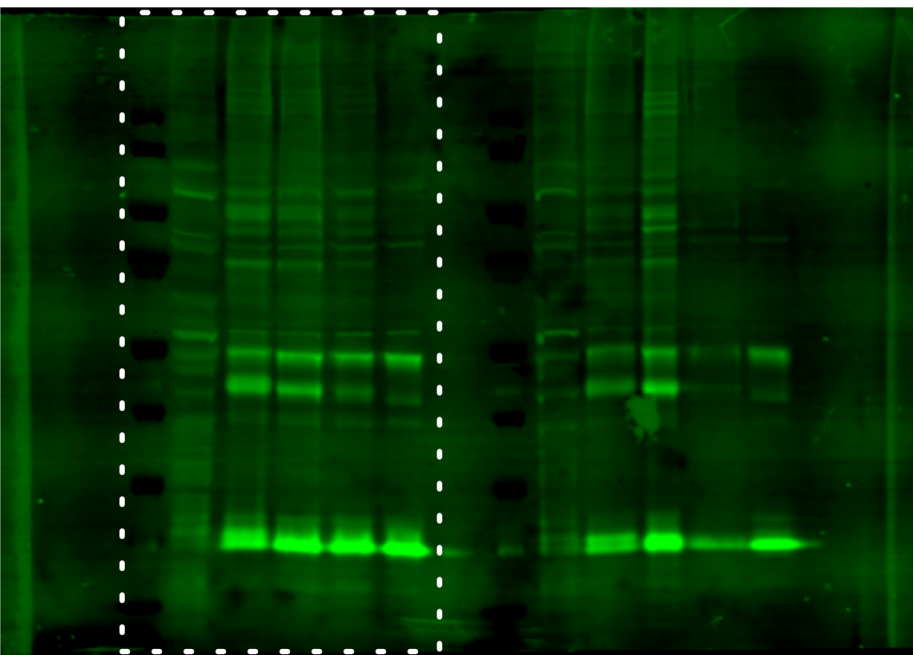

Supplement: Figure 3—source data 3. [file elife-88579-fig3-data3.zip › Figure 3ΓÇôSource Data 3.pdf]

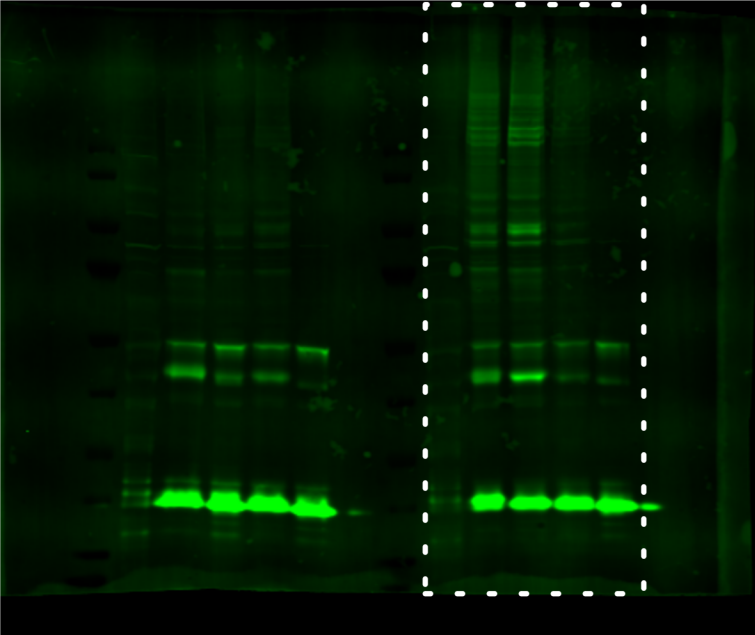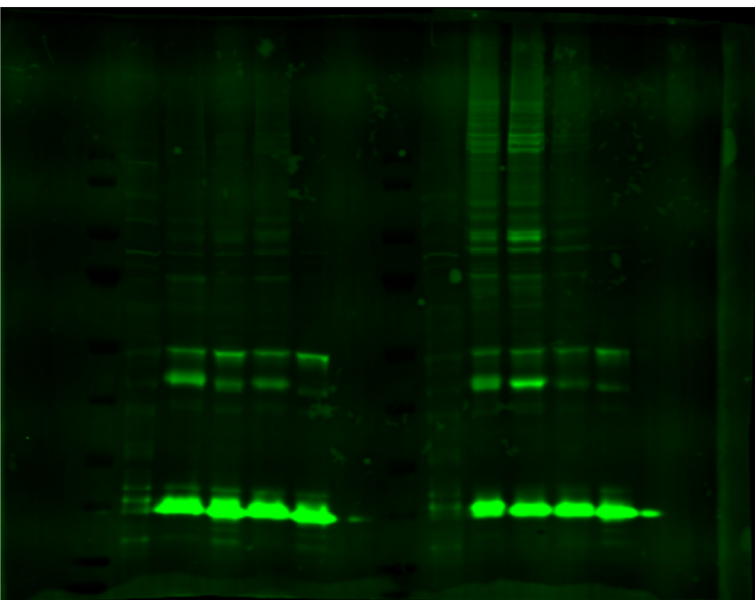

Supplement: Figure 3—source data 4. [file elife-88579-fig3-data4.zip › Figure 3ΓÇôSource Data 4.pdf]

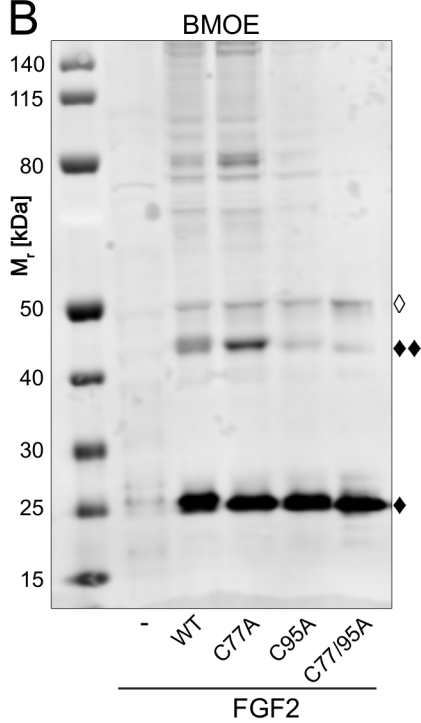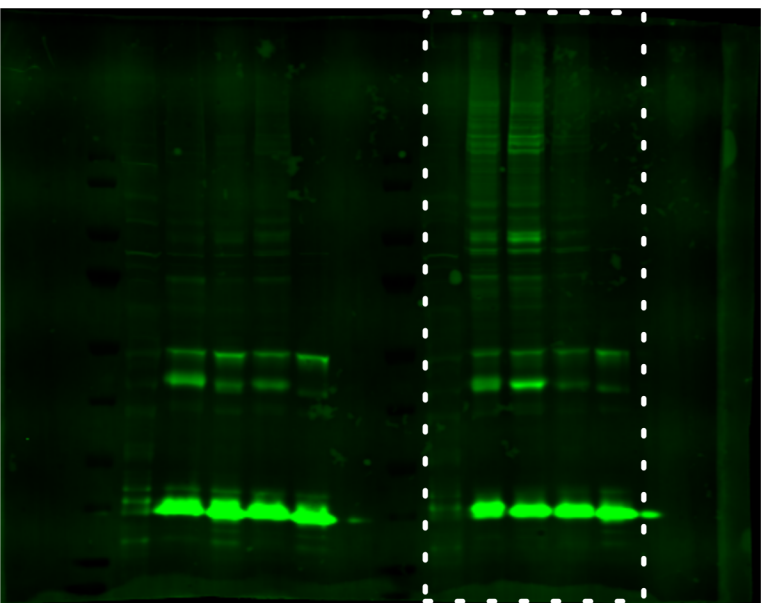

Supplement: Figure 3—source data 5. [file elife-88579-fig3-data5.zip › Figure 3ΓÇôSource Data 5.pdf]

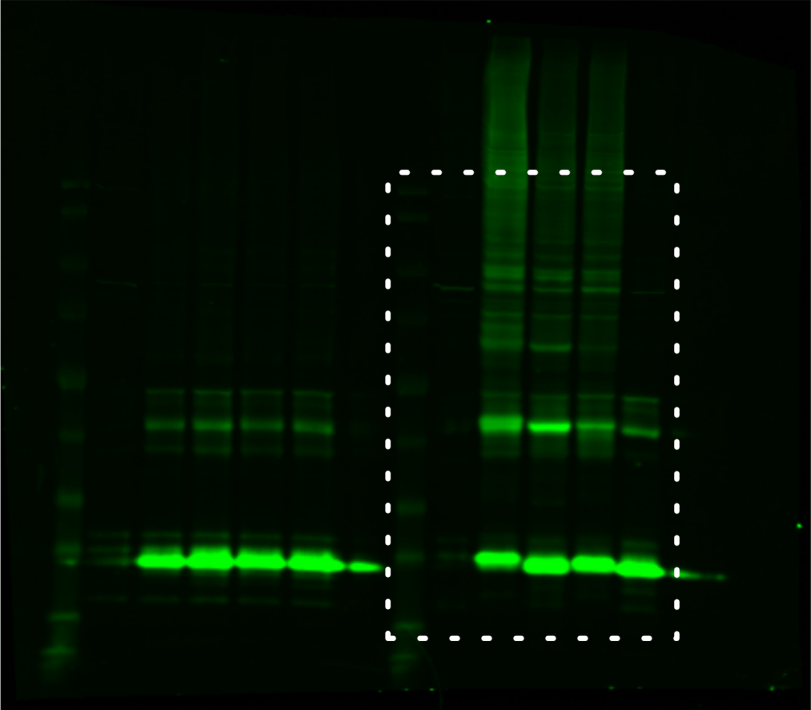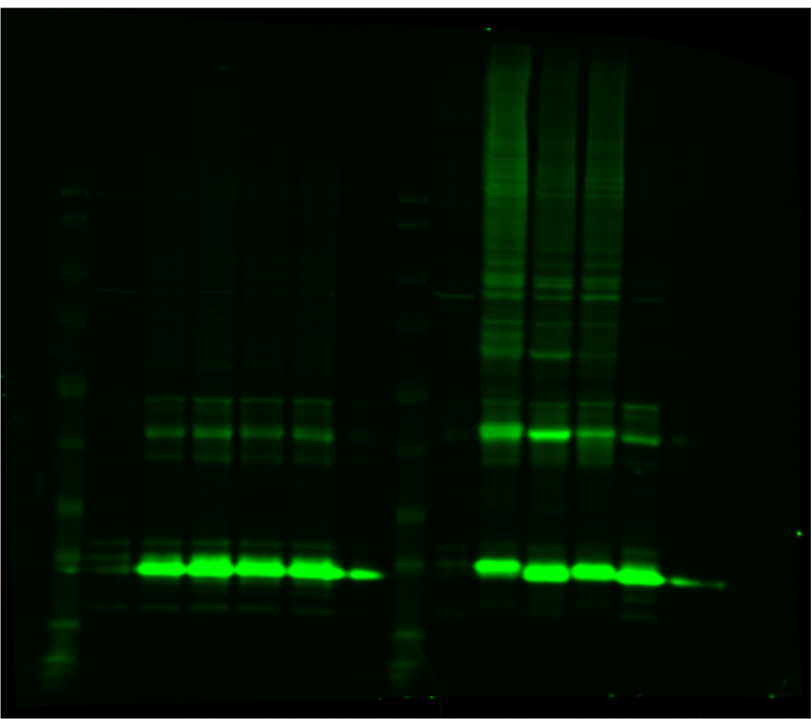

Supplement: Figure 3—source data 6. [file elife-88579-fig3-data6.zip › Figure 3ΓÇôSource Data 6.pdf]

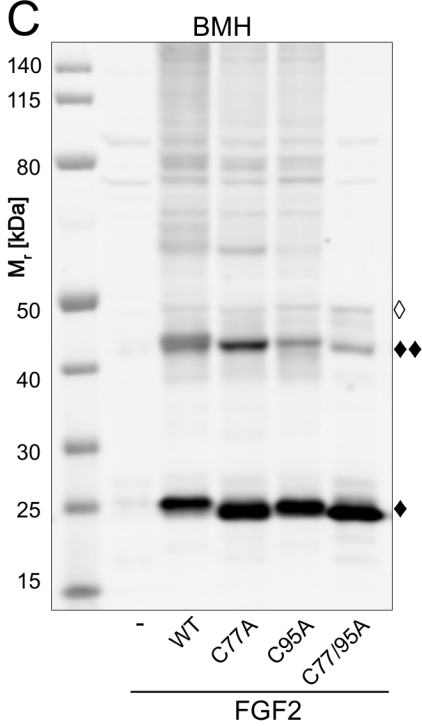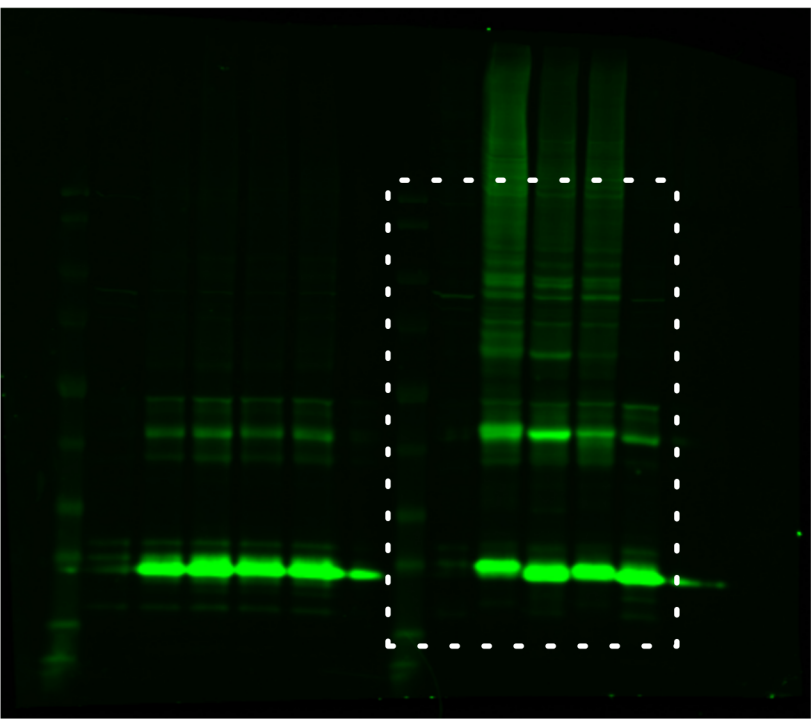

Supplement: Figure 3—source data 7. [file elife-88579-fig3-data7.zip › Figure 3ΓÇôSource Data 7.pdf]

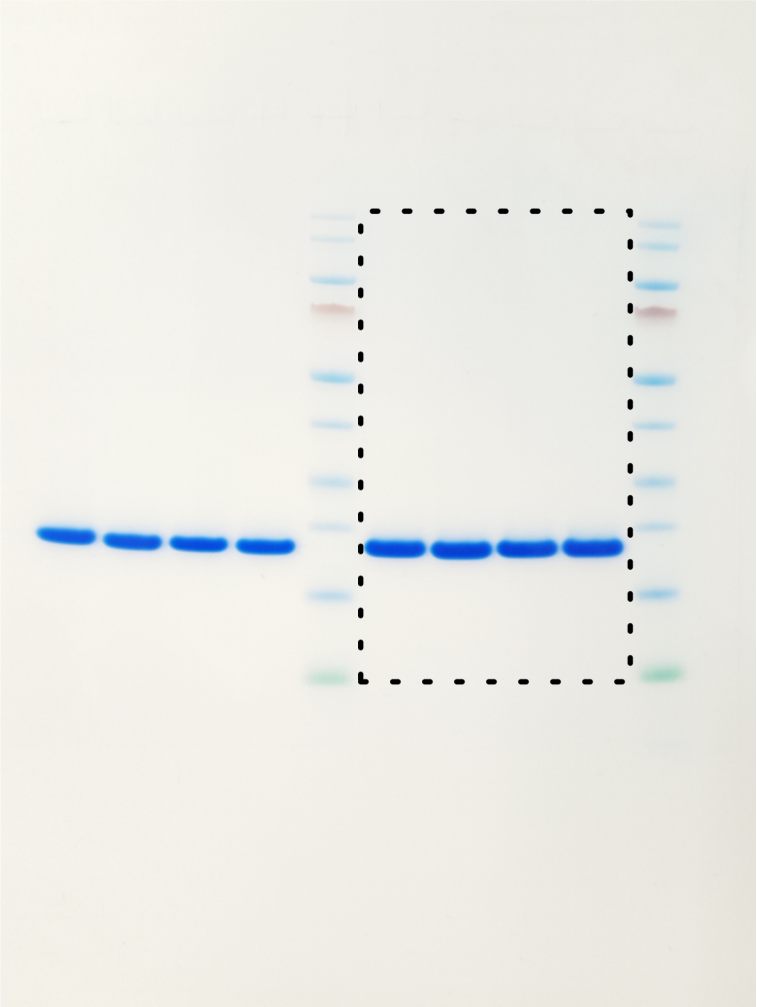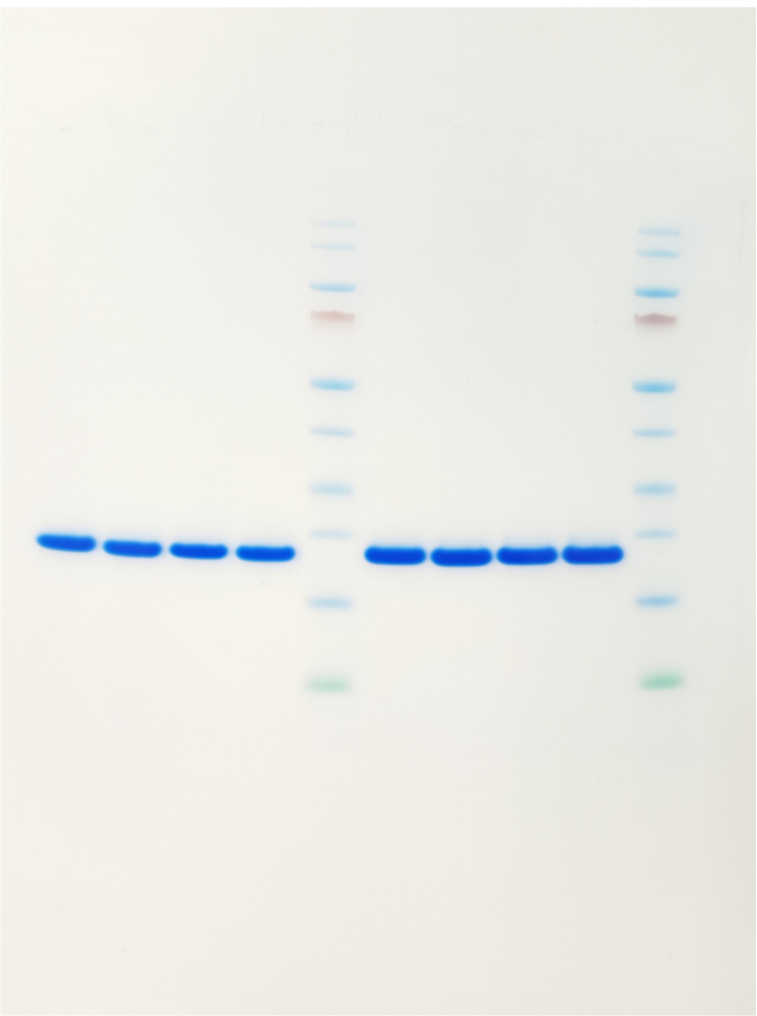

Supplement: Figure 4—source data 2. [file elife-88579-fig4-data2.zip › Figure 4ΓÇôSource Data 2.pdf]

**B**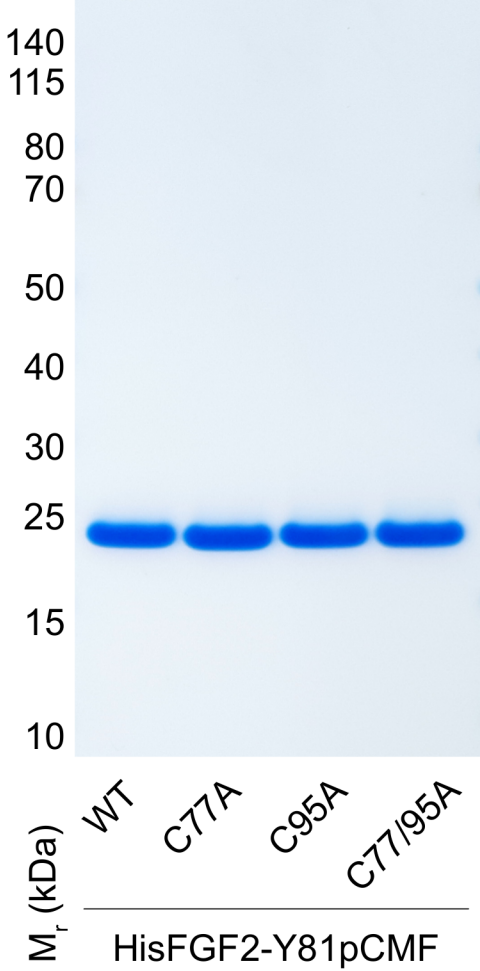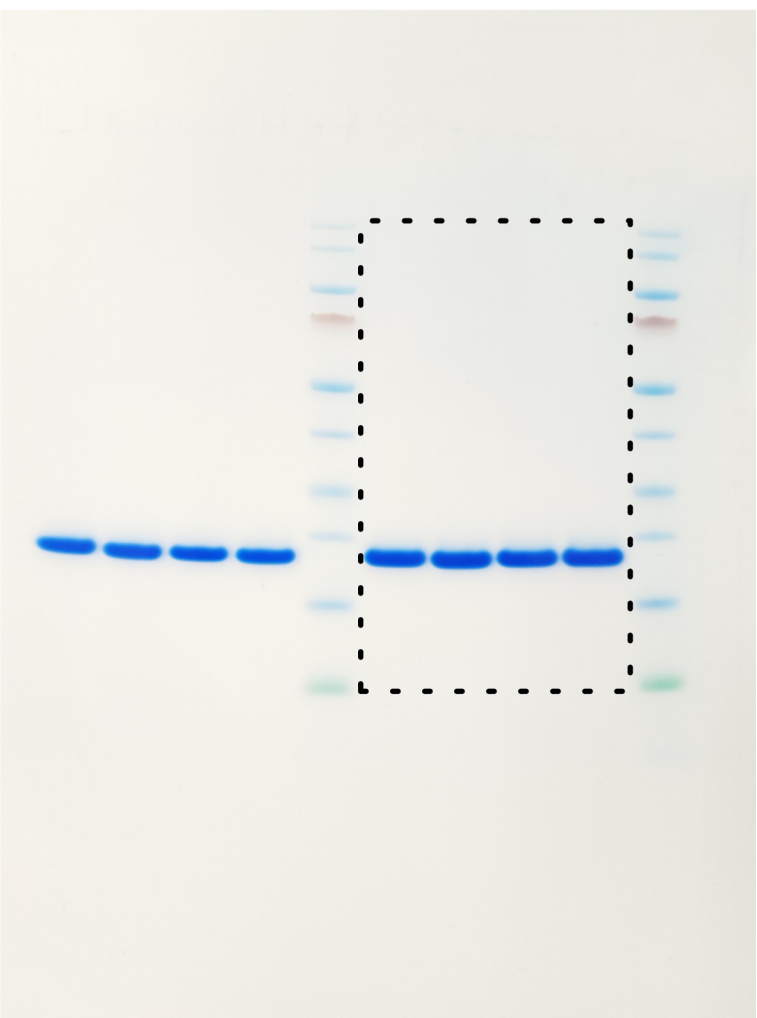

Supplement: Figure 4—source data 3. [file elife-88579-fig4-data3.zip › Figure 4ΓÇôSource Data 3.pdf]

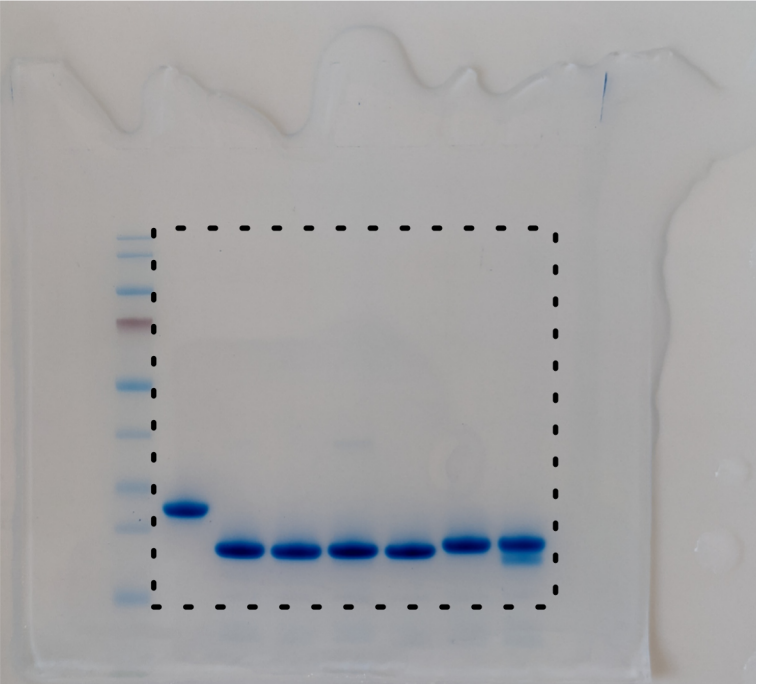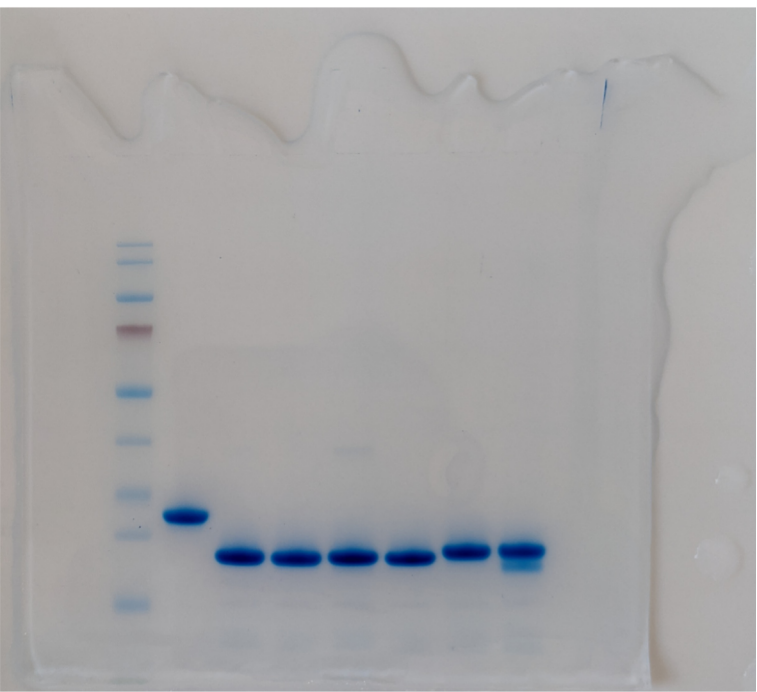

Supplement: Figure 7—source data 2. [file elife-88579-fig7-data2.zip › Figure 7ΓÇôSource Data 2.pdf]

D

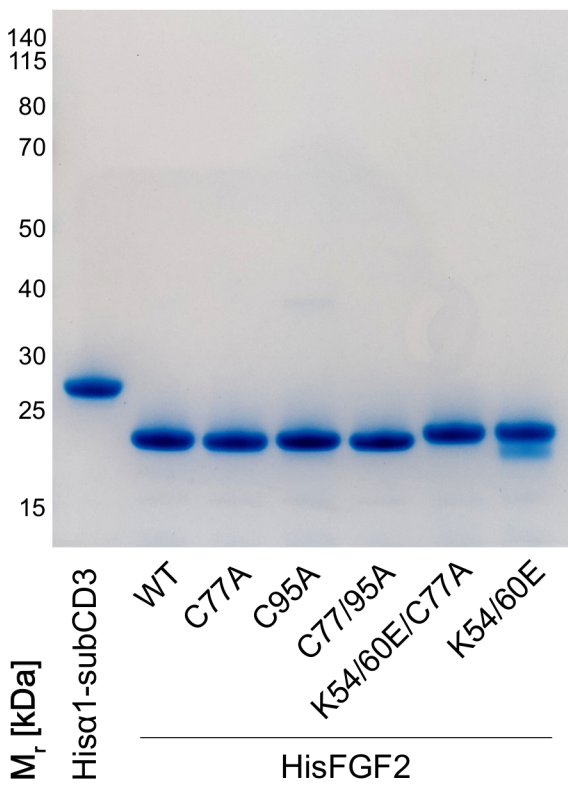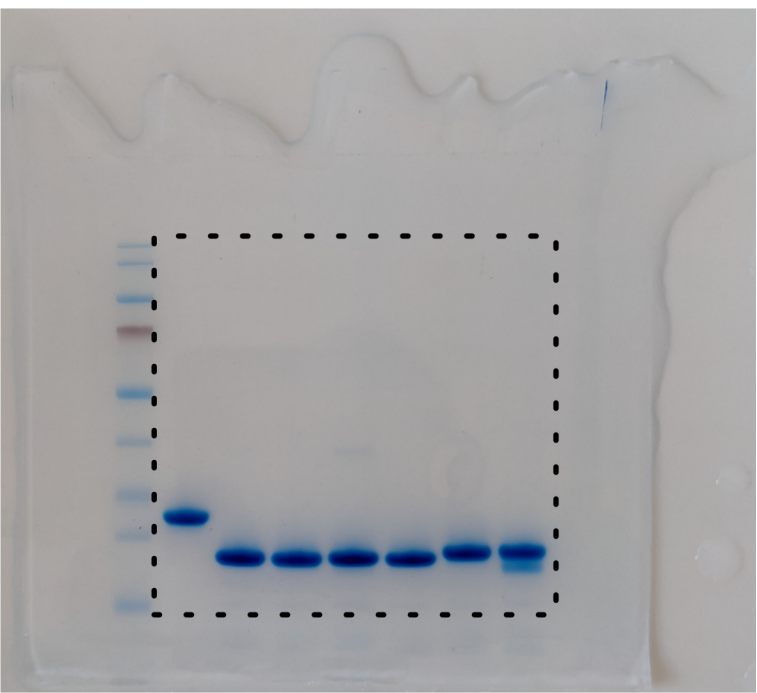

Supplement: Figure 7—source data 3. [file elife-88579-fig7-data3.zip › Figure 7ΓÇôSource Data 3.pdf]
